# Two-Dimensional Tetrahex-GeC_2_: A Material with Tunable Electronic and Optical Properties Combined with Ultrahigh Carrier Mobility

**DOI:** 10.1021/acsami.0c23017

**Published:** 2021-03-19

**Authors:** Wei Zhang, Changchun Chai, Qingyang Fan, Minglei Sun, Yanxing Song, Yintang Yang, Udo Schwingenschlögl

**Affiliations:** † School of Microelectronics, Xidian University, Xi’an 710071, China; ‡ College of Information and Control Engineering, Xi’an University of Architecture and Technology, Xi’an 710055, China; § Shaanxi Key Laboratory of Nano Materials and Technology, Xi’an 710055, China; ∥ Physical Science and Engineering Division (PSE), King Abdullah University of Science and Technology (KAUST), Thuwal 23955-6900, Saudi Arabia

**Keywords:** two-dimensional material, germanium carbide, narrow direct band gap semiconductor, band gap engineering, ultrahigh carrier mobility, first-principles calculations

## Abstract

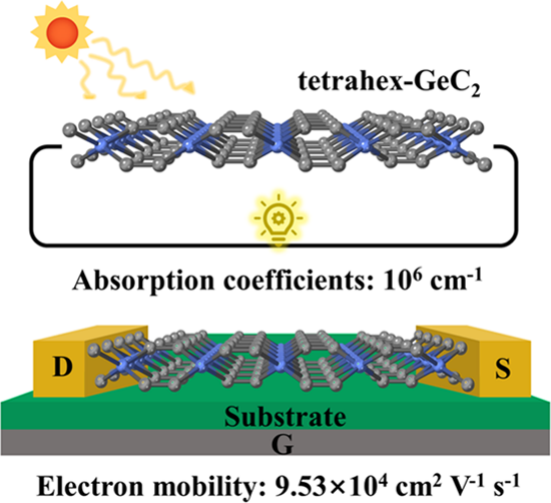

Based on first-principles calculations, we propose a novel two-dimensional (2D) germanium carbide, tetrahex-GeC_2_, and determine its electronic and optical properties. Each Ge atom binds to four C atoms, in contrast to the known 2D hexagonal germanium carbides. Monolayer tetrahex-GeC_2_ possesses a narrow direct band gap of 0.89 eV, which can be effectively tuned by applying strain and increasing the thickness. Its electron mobility is extraordinarily high (9.5 × 10^4^ cm^2^/(V s)), about 80 times that of monolayer black phosphorus. The optical absorption coefficient is ∼10^6^ cm^–1^ in a wide spectral range from near-infrared to near-ultraviolet, comparable to perovskite solar cell materials. We obtain high cohesive energy (5.50 eV/atom), excellent stability, and small electron/hole effective mass (0.19/0.10 *m*_0_). Tetrahex-GeC_2_ turns out to be a very promising semiconductor for nanoelectronic, optoelectronic, and photovoltaic applications.

## 1 Introduction

Since the first experimental realization of graphene,^1^ two-dimensional (2D) materials have attracted attention due to exotic structural and electronic properties.^2−5^ Belonging also to the class of group 14 2D materials, silicene^6^ and germanene^7^ show low buckled honeycomb structures and Dirac dispersions similar to graphene. Epitaxial growth has been achieved experimentally for both silicene^6,8−10^ and germanene.^7,11^ Many studies have demonstrated that group 14 2D materials are promising for next-generation nanoelectronic devices due to intriguing features such as high carrier mobility (massless Dirac fermions),^12^ significant spin–orbit coupling (which can induce band gaps of tens of meV),^13^ and extraordinary stiffness.^14^ However, the lack of adequate band gaps limits the applicability of graphene, silicene, and germanene in high-performance integrated logic circuits and optoelectronics, motivating the search for new 2D materials that combine a sizable band gap with high stability and good charge transport properties.

The fabrication of layered g-SiC with a thickness down to 0.5–1.5 nm has stimulated interest in binary 2D group 14 materials,^15−18^ as band gap opening has been predicted for this class of materials.^19^ Unlike silicene and germanene, the honeycomb structures of g-SiC and g-GeC are not buckled. Despite this structural similarity to graphene, g-SiC and g-GeC realize no massless Dirac fermions but sizeable band gaps. Monolayer g-SiC shows an indirect band gap of 2.56 eV,^19,20^ high exciton binding energies of up to 2.0 eV,^21^ and pronounced photoluminescence.^15^ Monolayer g-GeC exhibits a direct band gap of 2.19 eV and strong optical absorption in a wide spectral range.^22^ It has been demonstrated that the electronic and optical features of g-SiC and g-GeC nanosheets can be effectively tuned by introducing defects or adatoms, modifying the stacking sequence, adjusting the thickness, and applying strain or an external electric field.^23−26^ Numerous experimental and theoretical investigations have addressed the functionalization of g-SiC and g-GeC nanosheets for optoelectronic devices,^15,21,24,25,27^ integrated nanodevices,^28^ and metal-free electrocatalysts.^29^ Since photovoltaic applications are hindered by wide and indirect band gaps, extensive research efforts have been made toward predicting new 2D silicon carbides.^30−37^ For example, based on the density functional theory, Li et al.^30^ and Zhou et al.^31^ have reported metallic silagraphene and semiconducting siligraphene (g-SiC_2_) with a direct band gap of 1.09 eV, respectively. Li et al.,^32^ Shi et al.,^34^ and Borlido et al.^36^ have comprehensively studied the stabilities and electronic properties of monolayer Si_*x*_C_*y*_ for different stoichiometric compositions. On the other hand, so far no additional stable structures of 2D germanium carbides have been identified.

In this work, we predict a 2D germanium carbide (tetrahex-GeC_2_) that consists of tetra- and hexa-rings. The material exhibits excellent stability, an effectively tunable narrow direct band gap, ultrahigh electron mobility, and strong optical absorption in a wide spectral range, pointing to excellent potential in nanoelectronic, optoelectronic, and photovoltaic applications.

## 2 Theoretical Methods

First-principles calculations are performed using the Vienna ab initio simulation package (VASP),^38,39^ with the electron–ion interactions represented by projector-augmented wave pseudopotentials and the exchange-correlation potential described by the Perdew–Burke–Ernzerhof functional in the structure optimizations. The electronic band structure (including carrier effective mass and mobility) is obtained by the hybrid Heyd–Scuseria–Ernzerhof^40^ functional. The G_0_W_0_ approach^41^ is applied together with the Bethe–Salpeter equation^42,43^ for calculating accurate optical absorption spectra. The energy cutoff of the wave function expansion is set to 500 eV and a 9 × 9 × 1 Monkhorst–Pack mesh is used to sample the Brillouin zone. In calculations for multilayer tetrahex-GeC_2_, the DFT-D3 correction is used. All 2D models contain a 20 Å thick vacuum layer. We employ convergence tolerances of 1 × 10^–6^ eV/atom for the total energy and 0.001 eV/Å for the maximum atomic force. Phonon spectra are calculated by real-space density functional perturbation theory as implemented in VASP. The Phonopy code^44^ is used to calculate second-order force constants and phonon frequencies. Ab initio molecular dynamics (AIMD) simulations are carried out for 5 ps with a time step of 1 fs using a canonical ensemble.^45^

## 3 Results and Discussion

**Figure 1:**
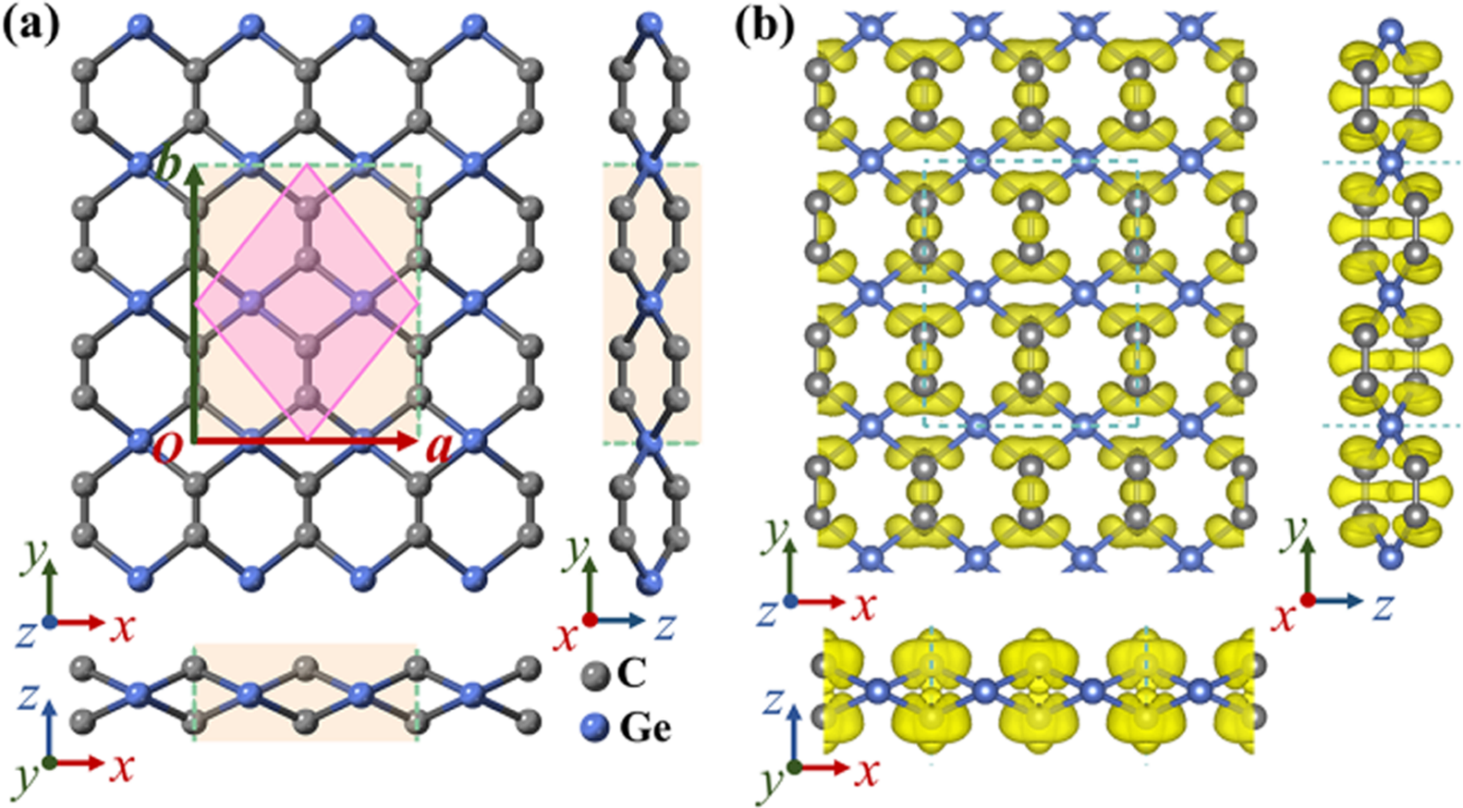
Monolayer tetrahex-GeC_2_: (a) Top and side views of the optimized atomic structure. The conventional and primitive cells are shown in yellow and pink, respectively. (b) ELF for an isosurface value of 0.75.

The optimized atomic structure of monolayer tetrahex-GeC_2_ is depicted in Figure 1a, with the Ge and C atoms represented by blue and gray spheres, respectively. The lattice is orthorhombic with the space group *Cmma* (No. 67) and the structure consists of a network of tetra- and hexa-rings. The conventional cell contains four Ge atoms and eight C atoms, with optimized lattice parameters of *a* = 5.89 Å and *b* = 7.29 Å. Each Ge atom bonds covalently to four C atoms with a bond length of 2.00 Å, which is 0.13 Å longer than the bond length of g-GeC.^46^ Each C atom bonds covalently with two Ge atoms and one C atom. A C–C bond length of 1.34 Å falls between the bond lengths of acetylenic linkage (1.20 Å) and graphene (1.42 Å). As the Ge atoms aim for their standard tetrahedral configuration, the structure is buckled with a total layer thickness of 1.41 Å. C–Ge–C bond angles of 85.1 and 109.4° demonstrate distortions as compared to bulk c–Ge (a bond angle of 109.5°). The Ge–C–Ge and Ge–C–C bond angles are 94.9 and 125.3°, respectively. Figure S1 summarizes the structural details of monolayer tetrahex-GeC_2_. While the structure is hardly affected by H_2_O in air, it turns out that monolayer tetrahex-GeC_2_ is susceptible to oxidation by breaking of C–Ge bonds and formation of C–O and Ge–O bonds (Figure S2). To analyze the chemical bonding, the electron localization function (ELF) is addressed in Figure 1b. High values at the bond centers suggest strong covalent C–C bonding, while asymmetric shapes suggests polar covalent Ge–C bonding (Bader charge imbalance of ∼0.65 electrons) in agreement with the smaller electronegativity of Ge than C.

**Figure 2:**
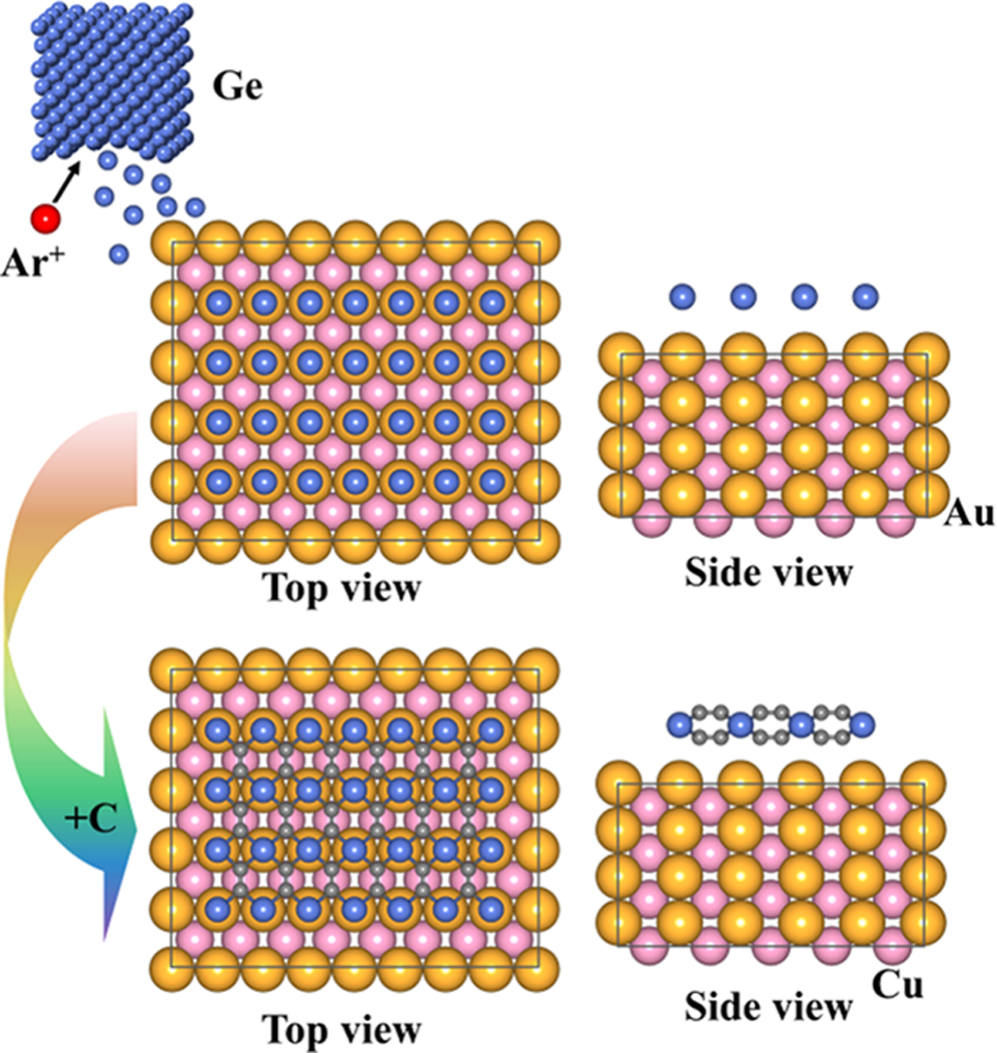
Synthesis strategy for tetrahex-GeC_2_ on the CuAu (110) surface using a Ge evaporator and annealing.

To verify the structural stability, we analyze the cohesive energy
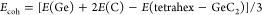
1where *E*(Ge) and *E*(C) are the total energies of isolated Ge and C atoms, respectively. With an obtained value of 5.50 eV/atom, monolayer tetrahex-GeC_2_ falls short of graphene (7.85 eV/atom) and h-BN (7.07 eV/atom) but outperforms 2D MoS_2_ (5.02 eV/atom), g-GeC (4.90 eV/atom), and Cu_2_Si (3.46 eV/atom),^47^ verifying high stability and great promise for future synthesis. More specifically, the lattice parameters of tetrahex-GeC_2_ (*a* = 5.89 Å and *b* = 7.29 Å) are close to those of a 2 × 2 supercell of the CuAu (110) surface (*a* = 5.60 Å and *b* = 7.34 Å).^48^ This fact enables synthesis by the strategy illustrated in Figure 2 since we find the strain experienced by tetrahex-GeC_2_ during this process to increase its cohesive energy by only 20 meV per atom. Deposition of Ge on the CuAu (110) surface is possible using a Ge evaporator by Ar^+^ ion bombardment and annealing, analogous with the synthesis of germanene on the Au(111) surface^7^ and silicene on the Ag(111) surface,^8^ with the C provided by ethylene or CaC_2_.^49,50^

**Figure 3:**
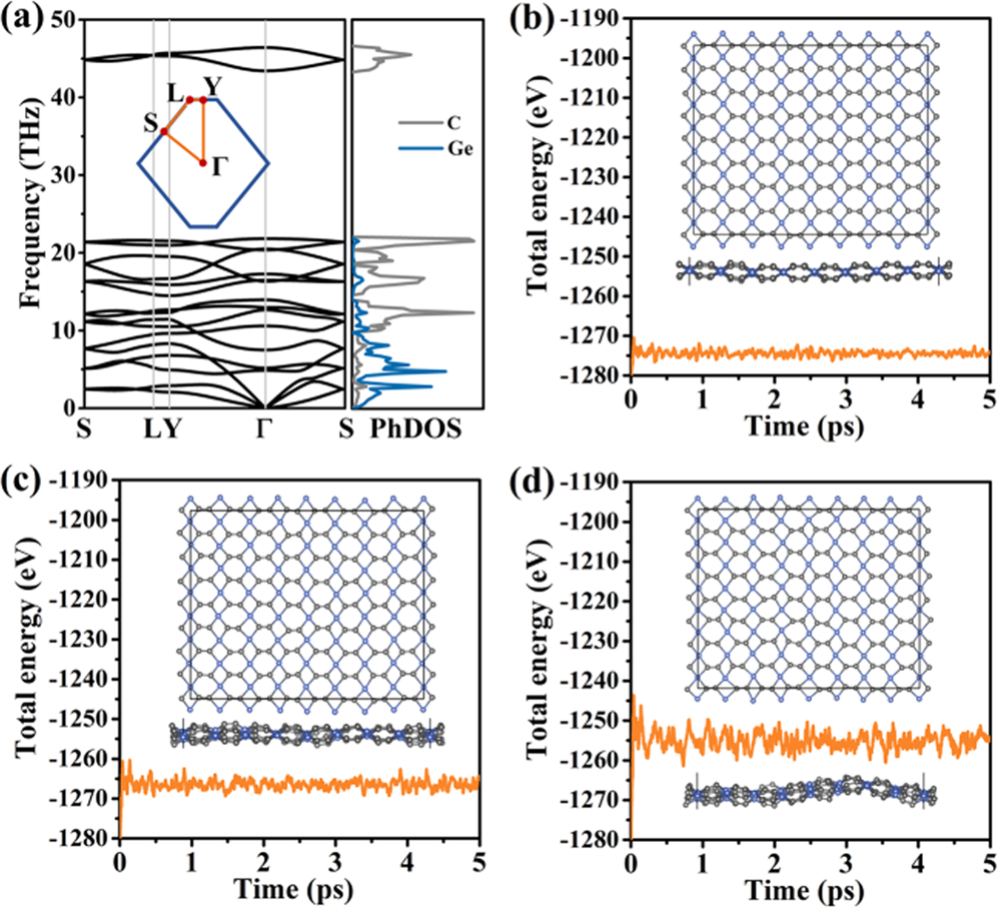
Monolayer tetrahex-GeC_2_: (a) Phonon band structure along high-symmetry directions of the 2D Brillouin zone (shown as the inset; primitive cell) and partial phonon densities of states. Fluctuations of the total energy during the AIMD simulations at (b) 300, (c) 600, and (d) 1000 K. The insets show the final atomic structures.

The phonon band structure and partial densities of states given in Figure 3a demonstrate the absence of imaginary phonon modes and, thus, dynamical stability of monolayer tetrahex-GeC_2_. The two highest optical branches fall in a frequency range of 43–46 THz and originate entirely from the vibrations of the sp^2^-hybridized C atoms. The final atomic structures after AIMD simulations at 300, 600, and 1000 K (4 × 4 supercell of the conventional cell) are presented in Figure 3b–d together with the fluctuations of the total energy. Neither C–C nor Ge–C bonds break during the simulations, nor are phase transitions observed. We find no significant structural distortions at 300 and 600 K. While monolayer tetrahex-GeC_2_ loses its planarity at 1000 K, the atomic skeleton is well preserved, indicating excellent thermal stability at least up to 1000 K.

**Figure 4:**
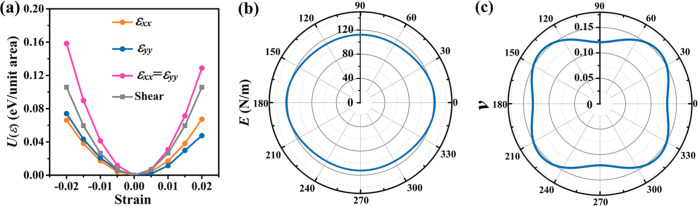
Monolayer tetrahex-GeC_2_: (a) Elastic energy under uniaxial, biaxial, and shear strains. In-plane angular dependence of (b) Young’s modulus and (c) Poisson’s ratio.

The in-plane elastic constants *C*_11_, *C*_22_, *C*_12_, and *C*_44_ of a 2D material are given by the second partial derivatives of the elastic energy^51^
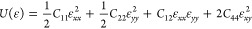
2see the results in Figure 4a. We obtain *C*_11_ = 124, *C*_22_ = 114, *C*_12_ = 15, and *C*_44_ = 49 N/m, which satisfy the mechanical stability criteria *C*_44_ > 0 and *C*_11_*C*_22_ – (*C*_12_)^2^ > 0. The in-plane Young’s modulus (*E*) and Poisson’s ratio (*v*) of a 2D material along an arbitrary direction θ (angle relative to the *x*-direction) are defined as^51^
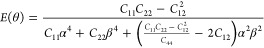
3and
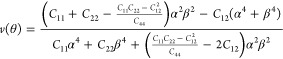
4where α = sin θ and β = cos θ. The results in Figure 4b,c evidence mechanical anisotropy due to the orthorhombic lattice. The maximum of Young’s modulus appears in the *x*-direction (122 N/m) and the minimum in the *y*-direction (112 N/m). Poisson’s ratios of *v*_*x*_ = 0.13 and *v*_*y*_ = 0.12 suggest that under tensile strain, the material expands less along the *x* than the *y*-direction.

**Figure 5:**
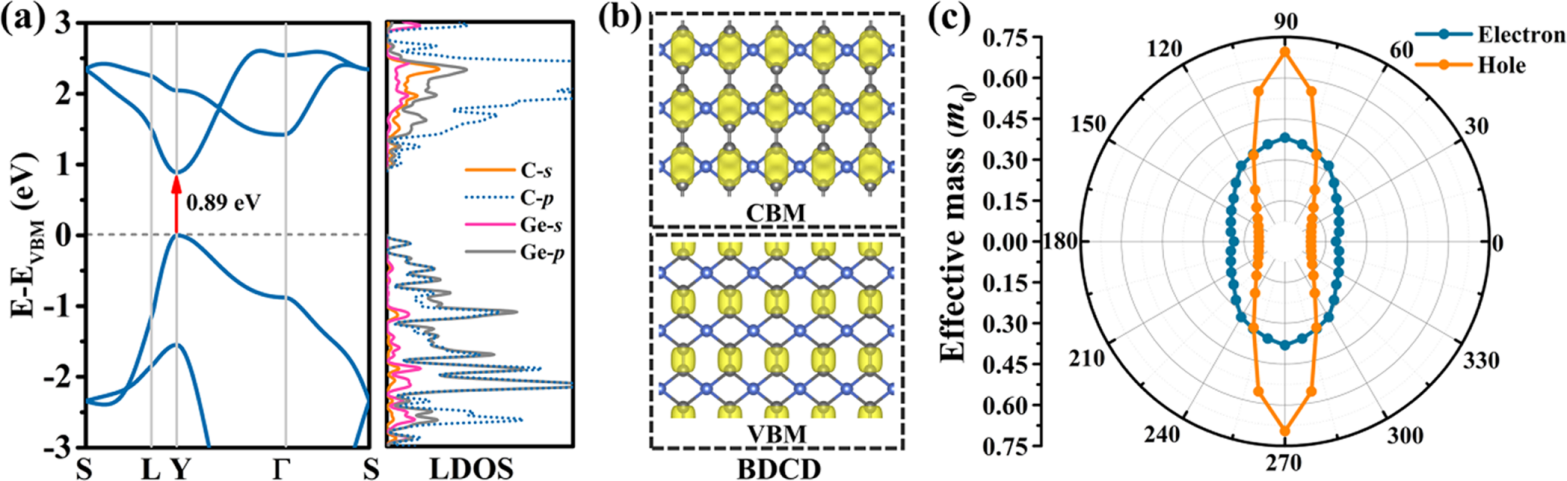
Monolayer tetrahex-GeC_2_: (a) Electronic band structure and partial densities of states. (b) Band-decomposed charge densities at the VBM and the CBM. The isosurface value is 0.02 eV/Å^3^. (c) Orientation-dependent carrier effective mass. The angle refers to the *x*-direction.

To analyze the electronic characteristics, we study the band structure and partial densities of states in Figure 5a. Monolayer tetrahex-GeC_2_ turns out to be a direct band gap semiconductor with a band gap of 0.89 eV. The conduction band minimum (CBM) and the valence band maximum (VBM) are located at the *Y* = (0.5, 0.5, 0) point. The partial densities of states suggest that the CBM is dominated by the C-p orbitals and the VBM by the C-p and Ge-p orbitals. The band-decomposed charge densities in Figure 5b show that the electronic states at the band edges are spatially localized at the sp^2^-hybridized C atoms. The hole and electron effective masses (direction *d*) are calculated by fitting the band edge dispersions (VBM and CBM, respectively) as
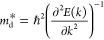
5The results in Figure 5c show strong anisotropy with the electron/hole effective mass being minimal in the *x*-direction (0.19/0.10 *m*_0_) and maximal in the *y*-direction (0.37/0.70 *m*_0_). The effective masses vary by factors of 2 (electrons) and 7 (holes), respectively. While the hole effective mass is smaller than the electron effective mass in the *x*-direction, the opposite applies to the *y*-direction. The obtained values are comparable with those of monolayer black phosphorus.^52^

**Figure 6:**
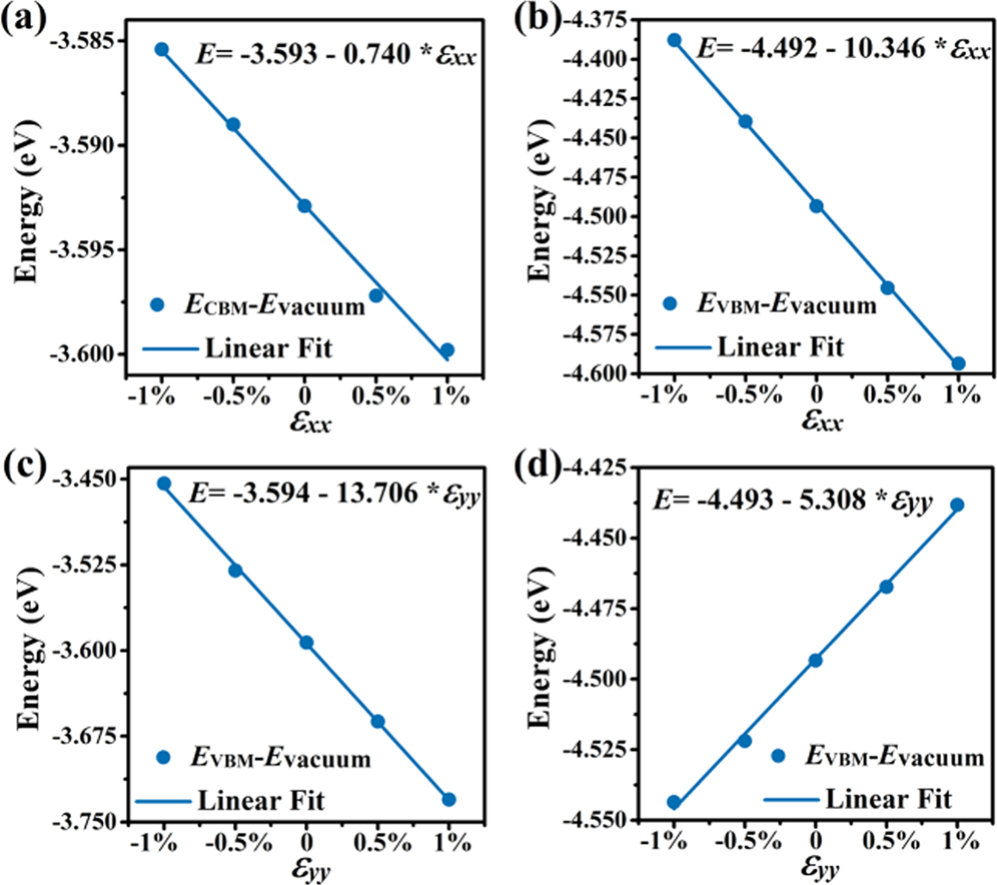
Monolayer tetrahex-GeC_2_: (a, c) *E*_VBM_ and (b, d) *E*_CBM_ relative to the vacuum energy *E*_vacuum_ as functions of small uniaxial strain along the (a, b) *x*- and (c, d) *y*-directions.

The low carrier effective masses point to high carrier mobilities
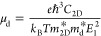
6where *C*_2D_ is the in-plane elastic constant, , and the deformation potential constant *E*_1_ is given by the shift of the band edge under strain. The band edge positions *E*_VBM_ and *E*_CBM_ relative to the vacuum energy *E*_vacuum_ are shown in Figure 6a–d as functions of strain and the obtained parameters at room temperature are summarized in Table 1. Both the deformation potential constants and carrier mobilities show significant anisotropy. We find that the electron mobility in the *x*-direction is almost 700 times that in the *y*-direction, which is a consequence of the very low effective mass and deformation potential constant (weak electron–phonon scattering). The hole mobility is also larger in the *x* than in the *y*-direction, by a factor of ∼2, due to the lower effective mass. The fact that the predicted electron mobility of monolayer tetrahex-GeC_2_ is more than 80 times that of monolayer black phosphorus (1100–1140 cm^2^/(V s))^52^ and the predicted hole mobility clearly exceeds that of monolayer MoS_2_ (200–270 cm^2^/(V s))^53,54^ suggests potential in high-performance electronic devices.

**Table 1:** Carrier Effective Mass, Deformation Potential Constant, In-Plane Elastic Constant, and Carrier Mobility at 300 K

carrier type	*d*	*m*_d_* (*m*_0_)	*E*_1_ (eV)	*C*_2D_ (N/m)	μ_d_ (cm^2^/(V s))
electron	*x*	0.19	0.74	124	9.53 × 10^4^
electron	*y*	0.37	13.71	114	132
hole	*x*	0.10	10.35	124	934
hole	*y*	0.70	5.31	114	466

**Figure 7:**
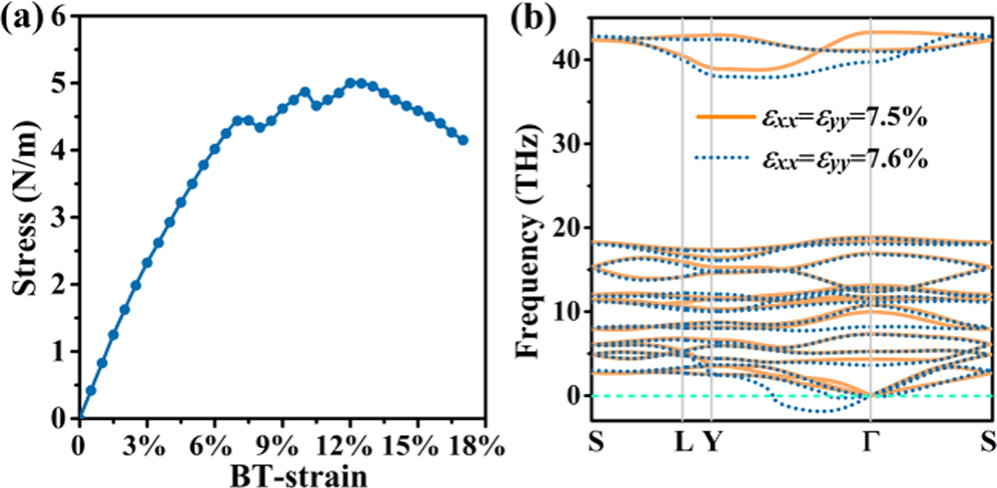
Monolayer tetrahex-GeC_2_: (a) Stress–strain relationship under biaxial tensile strain (obtained using the conventional cell). (b) Phonon band structure at 7.5 and 7.6% biaxial tensile strain.

**Figure 8:**
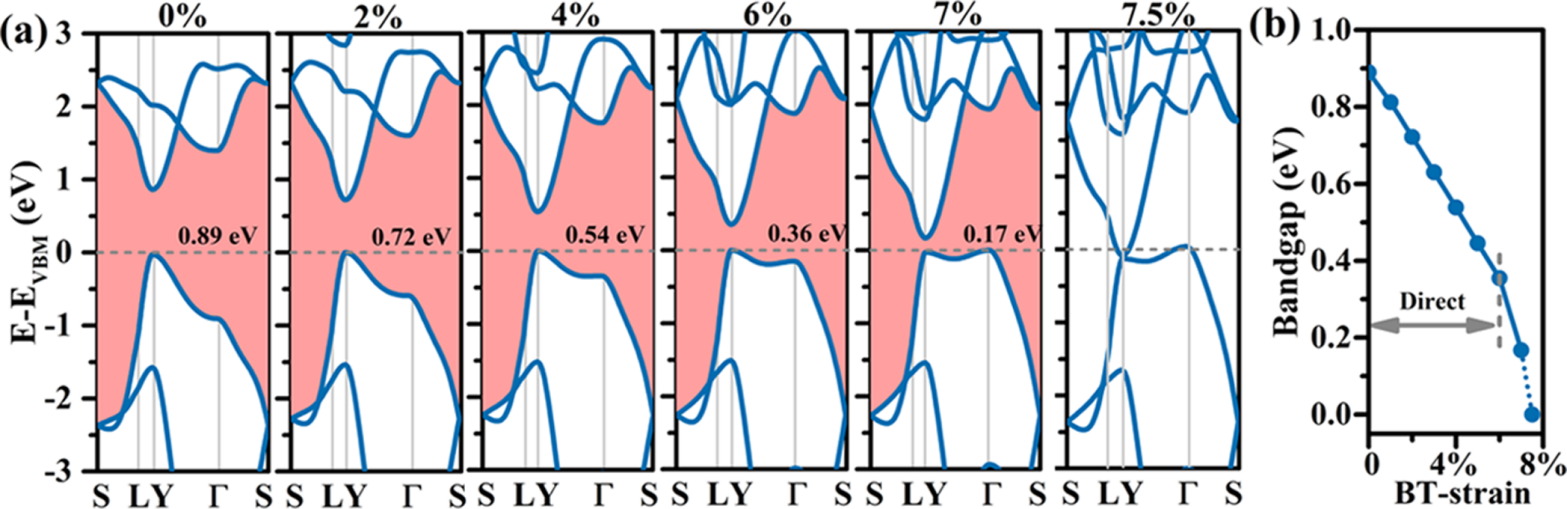
Monolayer tetrahex-GeC_2_: (a) Electronic band structure under 0, 2, 4, 6, 7, and 7.5% biaxial tensile strain. (b) Band gap under biaxial tensile strain.

We next investigate the effect of biaxial tensile strain on monolayer tetrahex-GeC_2_. The stress–strain relationship in Figure 7a shows a maximal stress of 5.10 N/m at the 12% strain. According to Figure 7b, imaginary phonon modes are absent at 7.5% but not at 7.6% biaxial tensile strain, indicating that the lattice of monolayer tetrahex-GeC_2_ becomes unstable in this range. The effects of biaxial tensile strain on the band structure are addressed in Figure 8a, and for the band gap, the results are summarized in Figure 8b. Up to 6% biaxial tensile strain, the direct band gap decreases from 0.89 to 0.36 eV. This tunability of the band gap enables band gap engineering as required for optoelectronic applications. At 7% biaxial tensile strain, the band gap becomes indirect with the VBM now located at the Γ point and shortly afterward; at 7.5% biaxial tensile strain, monolayer tetrahex-GeC_2_ becomes metallic.

**Figure 9:**
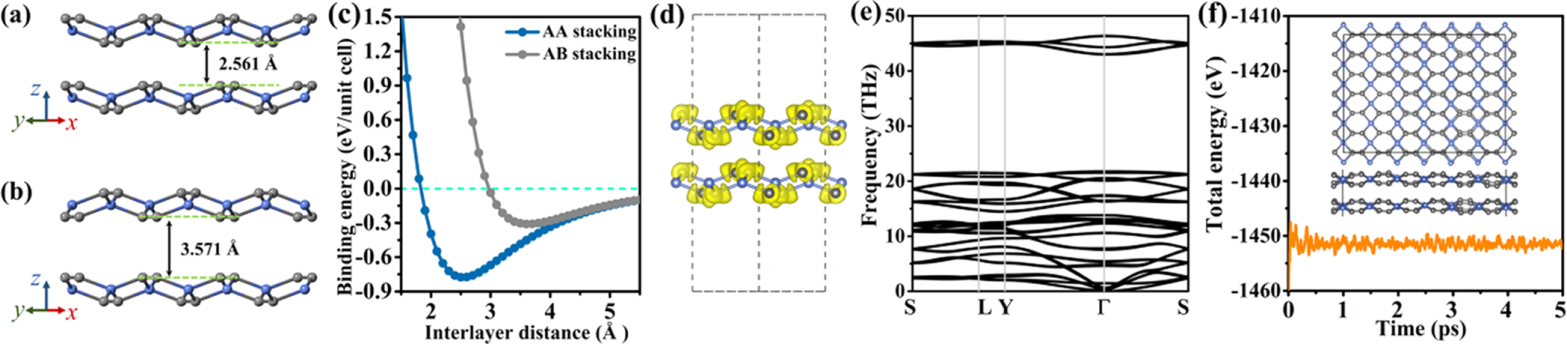
Bilayer tetrahex-GeC_2_: (a) AA stacking structure. (b) AB stacking structure. (c) Interlayer binding energies of the AA and AB stacking structures as functions of the interlayer distance. (d) ELF of the AA stacking structure for an isosurface value of 0.75. (e) Phonon band structure of the AA stacking structure. (f) Fluctuation of the total energy during the AIMD simulation of the AA stacking structure at 300 K. The inset shows the AA stacking structure after the simulation.

We further investigate bilayer tetrahex-GeC_2_ for AA stacking (Figure 9a; atomic positions identical in adjacent layers) and AB stacking (Figure 9b; atomic positions shifted by half of a lattice vector along the *x*- or *y*-direction). The interlayer binding energy (*E*_bilayer_ – 2*E*_monolayer_)/*A*, where *E*_bilayer_ and *E*_monolayer_ are the total energies of bilayer and monolayer tetrahex-GeC_2_, respectively, and *A* is the area, is shown in Figure 9c as a function of the interlayer distance (obtained with DFT-D3 correction by optimizing only the *x-* and *y*-coordinates of the atoms). With increasing interlayer distance, the interlayer binding energy changes from positive to negative, reaching for AA/AB stacking a minimum of −0.78/–0.31 eV per unit cell at a 2.5/3.6 Å interlayer distance. Full structure optimization for AA/AB stacking results in lattice parameters of *a* = 5.86/5.87 Å and *b* = 7.31/7.30 Å, an interlayer binding energy of −0.83/–0.35 eV per unit cell, and an interlayer distance of 2.56/3.57 Å. As it is energetically favorable, we study only AA stacking in the following. The ELF in Figure 9d agrees with the notion that the interlayer interaction is due to van der Waals forces and the phonon band structure in Figure 9e shows dynamic stability of bilayer tetrahex-GeC_2_ due to the absence of imaginary phonon modes. The results of AIMD simulations at 300 K (3 × 3 supercell of the conventional cell) in Figure 9f give no indication of bond breaking or phase transitions, indicating the thermal stability of bilayer tetrahex-GeC_2_.

**Figure 10:**
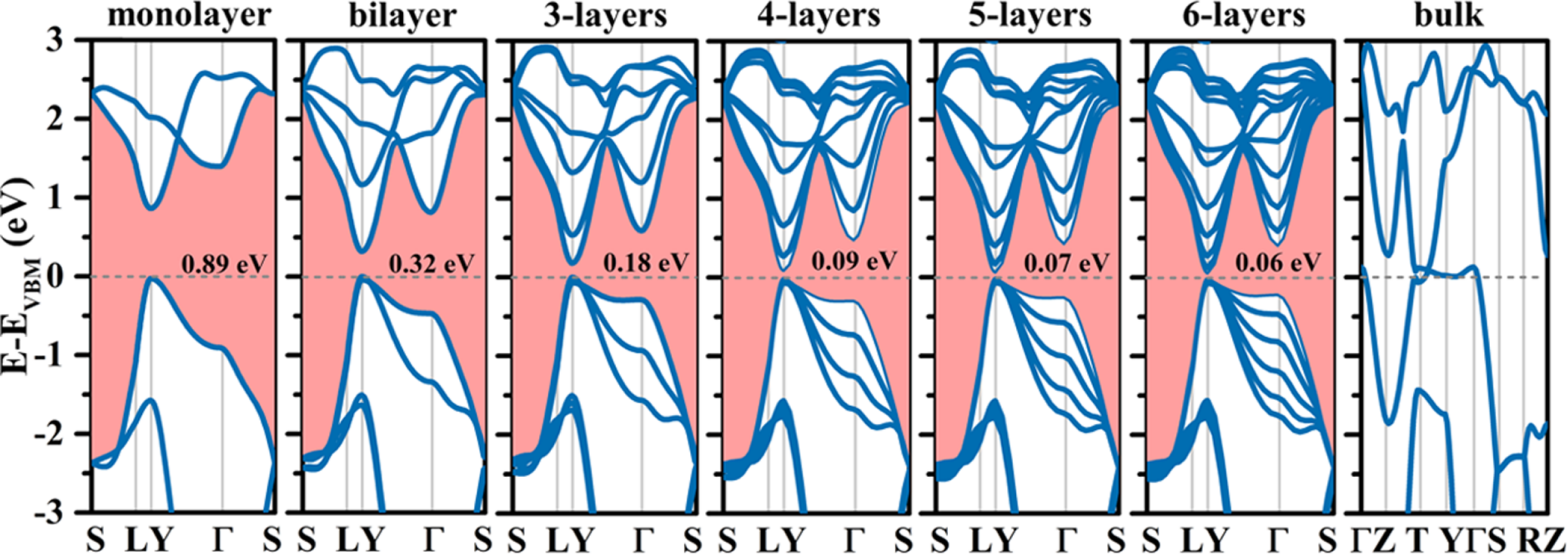
Electronic band structures of monolayer, bilayer, three-layer, four-layer, five-layer, six-layer, and bulk tetrahex-GeC_2_. The high-symmetry points in the Brillouin zone of bulk tetrahex-GeC_2_ are Γ = (0, 0, 0), *Z* = (0, 0, 0.5), *T* = (0.5, 0.5, 0.5), *Y* = (0.5, 0.5, 0), *S* = (0, 0.5, 0), and *R* = (0, 0.5, 0.5). Figure S3 shows the Brillouin zone of bulk tetrahex-GeC_2_.

Figure 10 shows the electronic band structures of monolayer, bilayer, three-layer, four-layer, five-layer, six-layer, and bulk tetrahex-GeC_2_ with AA stacking. The band gaps can be tuned from 0.89 eV in the case of monolayer tetrahex-GeC_2_ to 0.06 eV in the case of six-layer tetrahex-GeC_2_ without affecting the locations of the CBM and the VBM, i.e., the direct band gap is maintained. The band gap varies strongly from monolayer tetrahex-GeC_2_ to three-layer tetrahex-GeC_2_ and much less at higher thicknesses, which is explained by the fact that starting from three-layer tetrahex-GeC_2_, the structure contains bulk-like coordinated layers. Bulk tetrahex-GeC_2_ turns out to be metallic.

**Figure 11:**
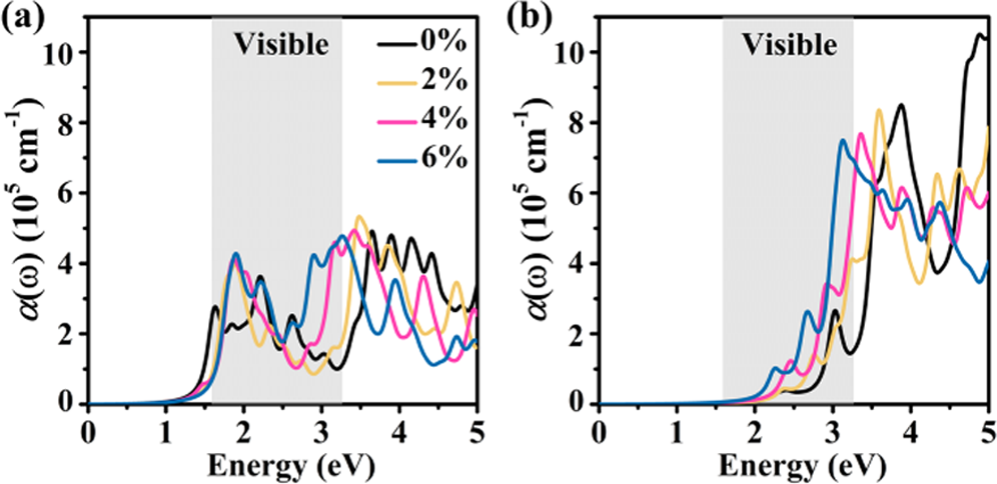
Monolayer tetrahex-GeC_2_: Optical absorption spectrum in the (a) *x-* and (b) *y*-directions under 0, 2, 4, and 6% biaxial tensile strain.

The fact that semiconductors with a narrow direct band gap can absorb light in the near-infrared and visible regions is key for applications in optoelectronics and photovoltaics. Figure 11 shows the optical absorption spectra of monolayer tetrahex-GeC_2_ under 0, 2, 4, and 6% biaxial tensile strain. For the *x*-direction, we find substantial absorption throughout the near-infrared, visible, and near-ultraviolet regions. For the *y*-direction, the absorption coefficient even reaches values of ∼10^6^ cm^–1^ in the near-ultraviolet region, comparable to perovskite solar cells.^55^ Mainly due to the shrinking band gap, biaxial tensile strain results in red shifts of the optical absorption spectra and promising enhancement of the absorption in the visible region.

## 4 Conclusions

We discover a novel 2D material, tetrahex-GeC_2_, and predict its mechanical, electronic, and optical properties. Great promise for future synthesis is demonstrated in terms of cohesive energy, phonon spectrum, thermal stability, stress–strain relationship, and elastic constants. Most notably, monolayer tetrahex-GeC_2_ shows extraordinarily high-room-temperature electron mobility as required for next-generation nanoelectronic devices. Its optical absorption coefficient in excess of 10^5^ cm^–1^ in the visible and near-ultraviolet regions is comparable to that of the perovskites currently employed in solar cells. Additionally, we find that biaxial tensile strain is able to substantially enhance the absorption of visible light, calling for the consideration of monolayer tetrahex-GeC_2_ in photovoltaics and optoelectronics. It turns out that the narrow direct band gap (0.89 eV) and small electron/hole effective mass (0.19/0.10 *m*_0_) of monolayer tetrahex-GeC_2_ can be effectively tuned by applying strain and/or by increasing the thickness to multilayer geometries. Our results thus offer a new strategy for achieving 2D germanium carbides with desirable material properties.

## Supporting Information Available

Structural details of tetrahex-GeC_2_; and coordinates and conventional cell of tetrahex-GeC_2_ (PDF)

## Abbreviations

2D: two-dimensional
AIMD: ab initio molecular dynamics
CBM: conduction band minimum
ELF: electron localization function
VASP: Vienna ab initio simulation package
VBM: valence band maximum
