# Supplementary material for: Two-Dimensional Tetrahex-GeC2: A Material with Tunable Electronic and Optical Properties Combined with Ultrahigh Carrier Mobility
Source: ACS Appl Mater Interfaces. 2021 Mar 19;13(12):14489–96. doi: 10.1021/acsami.0c23017 (PMC8041257; doi:10.1021/acsami.0c23017)
Supplement: Supplementary file 1 — am0c23017_si_001.pdf [file am0c23017_si_001.pdf]

Supporting Information

Two-Dimensional Tetrahex-GeC_2: A Material with Tunable Electronic and Optical Properties Combined with Ultrahigh Carrier Mobility

Wei Zhang,[*,†] Changchun Chai,[†] Qingyang Fan,[‡,§] Minglei Sun,[⁋] Yanxing Song,[†] Yintang Yang,[†] and Udo Schwingenschlögl[*,⁋]

[†]School of Microelectronics, Xidian University, Xi'an 710071, China.

[‡]College of Information and Control Engineering, Xi'an University of Architecture and Technology, Xi'an 710055, China.

[§]Shaanxi Key Laboratory of Nano Materials and Technology, Xi'an, 710055, China.

[⁋]Physical Science and Engineering Division (PSE), King Abdullah University of Science and Technology (KAUST), Thuwal 23955-6900, Saudi Arabia.

*W. Zhang (Email: wzhang-1993@stu.xidian.edu.cn)

*U. Schwingenschlögl (Email: udo.schwingenschlogl@kaust.edu.sa)

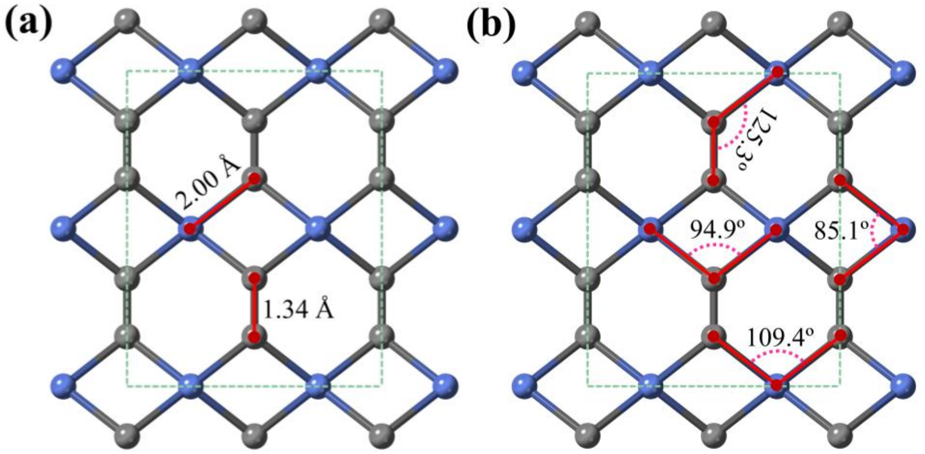

Figure S1. Monolayer tetrahex-GeC_2: (a) Bond lengths and (b) bond angles.

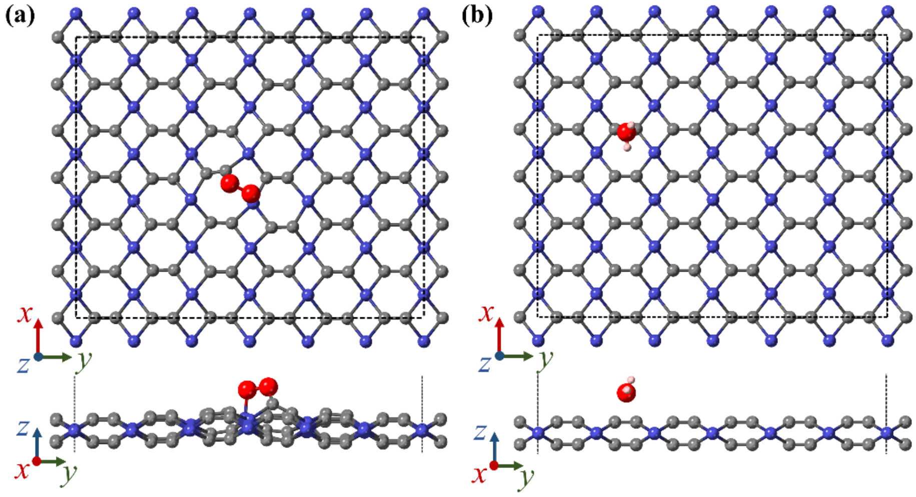

Figure S2. Top and side views of physisorbed (a) O_2 and (b) H_2O molecules on monolayer tetrahex-GeC_2. The pink, red, blue, and gray spheres represent H, O, Ge, and C atoms, respectively.

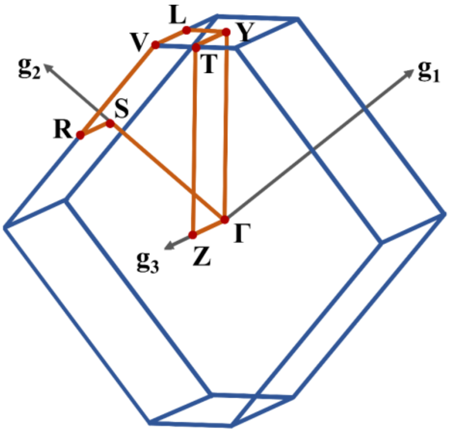

Figure S3. Brillouin zone of bulk tetrahex-GeC_2 with high symmetry points.

I. Atomic coordinates of monolayer tetrahex-GeC_2 in POSCAR format

```
Tetrahex-GeC2 monolayer
1.0
5.8871998787    0.0000000000    0.0000000000
0.0000000000    7.2944002151    0.0000000000
0.0000000000    0.0000000000    25.0000000000
C    Ge
8    4
Direct
0.500000000    0.841889957    0.471809998
0.500000000    0.158110026    0.528190002
0.500000000    0.658110043    0.471809998
0.500000000    0.341889990    0.528190002
0.000000000    0.341889990    0.471809998
0.000000000    0.658110043    0.528190002
0.000000000    0.158110026    0.471809998
0.000000000    0.841889957    0.528190002
0.250000000    0.500000000    0.500000000
0.750000020    0.500000000    0.500000000
0.750000020    0.000000000    0.500000000
0.250000000    0.000000000    0.500000000
```

II. Atomic coordinates of bilayer tetrahex-GeC_2 for AA stacking in POSCAR format

```
AA stacking structure
1.0
5.8578348748     0.0000000000      0.0000000000
0.0000000000     7.3144071809      0.0000000000
0.0000000000     0.0000000000     25.0000000000
C     Ge
16     8
Direct
0.500000000     0.841500050     0.270638972
0.500000000     0.158390423     0.326795894
0.500000000     0.658499949     0.270638972
0.500000000     0.341609560     0.326795894
0.500000000     0.341500017     0.270638972
0.500000000     0.658390407     0.326795894
0.500000000     0.158499966     0.270638972
0.500000000     0.841609593     0.326795894
0.500000000     0.841609592     0.429240082
0.500000000     0.158499966     0.485397005
0.500000000     0.658390407     0.429240082
0.500000000     0.341500017     0.485397005
0.000000000     0.341609560     0.429240082
0.000000000     0.658499950     0.485397005
0.000000000     0.158390423     0.429240082
0.000000000     0.841500050     0.485397005
0.250000000     0.500000000     0.298851282
0.749999939     0.500000000     0.298851282
0.749999939     0.000000000     0.298851282
0.250000000     0.000000000     0.298851282
0.250000000     0.500000000     0.457184713
0.749999939     0.500000000     0.457184713
0.749999939     0.000000000     0.457184713
0.250000000     0.000000000     0.457184713
```

III. Atomic coordinates of bilayer tetrahex-GeC_2 for AB stacking in POSCAR format

```
AB stacking structure
1.0
5.8679800162    0.0000000000     0.0000000000
0.0000000000    7.2974727609     0.0000000000
0.0000000000    0.0000000000    25.0000000000
C    Ge
16    8
Direct
0.500000000    0.841808777    0.250363453
0.500000000    0.158210041    0.306576461
0.500000000    0.658191222    0.250363453
0.500000000    0.341789959    0.306576461
0.000000000    0.341808777    0.250363453
0.000000000    0.658210073    0.306576461
0.000000000    0.158191222    0.250363453
0.000000000    0.841789926    0.306576461
0.000000000    0.841789926    0.449459457
0.000000000    0.158191222    0.505672522
0.000000000    0.658210073    0.449459457
0.000000000    0.341808777    0.505672522
0.500000000    0.341789959    0.449459457
0.500000000    0.658191222    0.505672522
0.500000000    0.158210041    0.449459457
0.500000000    0.841808777    0.505672522
0.250000000    0.500000000    0.278558934
0.749999959    0.500000000    0.278558934
0.749999959    0.000000000    0.278558934
0.250000000    0.000000000    0.278558934
0.749999959    0.500000000    0.477477080
0.249999939    0.500000000    0.477477080
0.249999939    0.000000000    0.477477080
0.749999959    0.000000000    0.477477080
```
